# Increased CA3 burst activity in *Doc2α* and *Syt7* knockout mice

**DOI:** 10.64898/2026.07.01.735713

**Published:** 2026-07-06

**Authors:** Raghava Jagadeesh Salaka, Edwin R. Chapman

**Affiliations:** 1 Department of Neuroscience, University of Wisconsin-Madison, Madison, WI, United States.; 2 Howard Hughes Medical Institute, Madison, WI, United States

**Keywords:** CA3, hippocampus, Doc2α, Syt7, CSBs, burst, recurrent collaterals

## Abstract

The hippocampal CA3 subfield is central to associative learning and memory consolidation. The principal cells of the CA3, pyramidal neurons, execute these functions by generating hypersynchronous bursts that feed forward to the CA1. Extensive recurrent collateral connections within the CA3 neuron population are crucial for the generation of this burst activity. Double C2 domain-containing protein α (Doc2α) and synaptotagmin 7 (Syt7) are high-affinity calcium sensors implicated in asynchronous synaptic vesicle (SV) release and in the exocytosis of dense-core vesicles (DCVs). Additionally, Doc2α is a sensor for miniature neurotransmission, whereas Syt7 is involved in synaptic facilitation and SV replenishment. Both Doc2α and Syt7 are expressed in the hippocampus, but their potential roles in spontaneous excitatory network activity remain unanswered. Using whole-cell recordings in disinhibited acute hippocampal slices obtained from juvenile *Doc2α-* and *Syt7-* knockout (KO) mice (P15–21), we report increased CA3 burst generation without changes in spontaneous excitatory postsynaptic current (sEPSC) frequency or amplitude. Moreover, the intrinsic properties of CA3 pyramidal neurons, such as the resting membrane potential, firing rate and input resistance, are unchanged. We propose that this novel burst phenotype in *Doc2α-* and *Syt7-* KO mice is unrelated to changes in SV release but might be mediated by changes in neuropeptide release from DCVs. Regardless of the underlying mechanisms, this work reveals that both proteins act to regulate network activity.

## Introduction

The hippocampal *cornu ammonis* 3 (CA3) region is critical for associative learning and memory. CA3 pyramidal neurons have axons with extensive recurrent collaterals (RCs) forming synapses within their own population (CA3→CA3) (Rebola et al., 2017; Joo & Frank, 2018). Under physiological conditions, RCs generate complex spike bursts (CSBs), a hallmark feature of CA3 pyramidal neurons. CSBs comprise two to six action potentials, with interspike intervals of < 6 ms, that progressively decrease in amplitude (Raus Balind et al., 2019). The cellular-level CSBs drive hypersynchronous network activity *in vivo*, termed sharp waves (SPWs), which, in turn, cause oscillatory activity called sharp wave ripples (SPW-Rs) in the CA1 subfield via the Schaffer collateral pathway (SC→CA1). The occurrence of SPWs or SPW-Rs is high during rest and non-rapid eye movement (NREM) sleep, and is thought to play an important role in reliable information transfer and memory consolidation (Buzsáki, 2015). However, increased CA3 burst activity is a major pathological finding in many neurological disorders, including epilepsy, and the CA3 subfield is a major seizure onset zone in temporal lobe epilepsy (Goldberg & Coulter, 2013).

Double C2 domain-containing protein 2ɑ (Doc2α) and synaptotagmin 7 (Syt7) are high-affinity calcium (Ca^2+^) sensors with similar, slow, intrinsic kinetics (Hui et al., 2005). Both proteins bind Ca^2+^ via their tandem C2 domains, C2A and C2B. Syt7 is a membrane protein (Vevea et al., 2021), whereas Doc2α is a cytosolic protein that translocates to the plasma membrane in response to Ca^2+^ influx (Xue et al., 2015). Doc2α and Syt7 play a role in the asynchronous release (AR) of neurotransmitters from nerve terminals (Yao et al., 2011; Xue et al., 2015; Chen et al., 2017; Turecek & Regehr, 2018; Deng et al., 2020; Wu et al., 2024). In neuroendocrine cells, both proteins serve as Ca^2+^ sensors that trigger dense-core vesicle (DCV) exocytosis and the release of neuropeptides and hormones (MacDougall et al., 2018; van Westen et al., 2021). In addition, Doc2α (and a closely related isoform, Doc2β) functions as a Ca^2+^ sensor that regulates spontaneous neurotransmitter release (Groffen et al., 2010; Courtney et al., 2018), and Syt7 regulates synaptic facilitation and SV replenishment (Chen et al., 2017; Jackman & Regehr, 2017).

Doc2α and Syt7 are expressed by CA3 neurons (Verhage et al., 1997; Jackman & Regehr, 2017). While progress has been made regarding the cellular functions of these proteins, their roles in network activity are unclear and remain controversial. In primary neuronal cultures, knockdown (KD) of *Doc2α* reduces reverberatory activity (Yao et al., 2011) while another study found no effect on burst activity (Ramirez et al., 2017). In brain slice preparations, increased spontaneous firing activity was seen in the Purkinje cells in *Doc2β* knockout (KO) mice (Groffen et al., 2010). However, another study reported reduced spontaneous action potential firing in the perforant pathway (EC→DG) in *Doc2α* KO mice (Wang et al., 2023). We therefore addressed the potential roles of Doc2α and Syt7 in regulating spontaneous excitatory network activity. Using both voltage-clamp and current-clamp approaches, we report increased CA3 burst generation in acute hippocampal slices in juvenile *Doc2α-* and *Syt7-* KO mice, following pharmacological disinhibition. The increased burst activity does not appear to be due to changes in neurotransmitter release mechanisms or the intrinsic properties of CA3 neurons, indicating a novel role for Doc2α and Syt7 in network activity. We propose that these two Ca^2+^ sensors affect burst generation by regulating the release of neuropeptides in the hippocampus.

## Methods

### Animals

*Doc2α* knockout (*Doc2α*^*−/−*^) (Sakaguchi et al., 1999), *Syt7* knockout (*Syt7*^*−/−*^) (Chakrabarti et al., 2003) and double knockout (DKO) (*Doc2α*^*−/−*^*/Syt7*^*−/−*^) (Wu et al., 2024) mice were bred as homozygous pairs. Detailed information regarding these mouse lines have been described previously (Wu et al., 2024). All mice were housed in polycarbonate cages on a 12h dark/light cycle and with food and water provided *ad libitum*. We did not observe any sex differences; therefore, data from both sexes were pooled. Animal experiments were performed in accordance with the National Institutes of Health guidelines for animal research and were approved by the Animal Care and Use Committees (IACUC, assurance number: A3368–01) at the University of Wisconsin-Madison.

### Acute slice preparation

P15–21 mice were decapitated under isoflurane anesthesia. Brains were quickly chilled in ice-cold sucrose-based cutting solution containing (in mM): 210 sucrose, 2.5 KCl, 25 NaHCO_3_, 1.25 NaH_2_PO_4_, 20 glucose, 7 MgSO_4_ 7H_2_O and 0.5 CaCl_2_ 2H_2_O. Two minutes later, hemispheres were isolated, and modified transverse hippocampal slices were cut at 300 μm thickness using the 10° angle blocking cut (Bischofberger et al., 2006) with a vibratome (VT1200s, Leica Microsystems) in the same solution. Slices were transferred to an incubator (BSK1, Automate Scientific) filled with prewarmed artificial cerebrospinal fluid (aCSF) (~34°C) for 20 min and then stored at room temperature until recording (6–8 h). The aCSF contained the following components (in mM): 125 NaCl, 2.5 KCl, 25 NaHCO_3_, 1.25 NaH_2_PO_4_, 20 glucose, 1 MgSO_4_ 7H_2_O, and 1.2 CaCl_2_ 2H_2_O. The osmolarity of the aCSF was measured using a vapor pressure osmometer (VAPRO^®^ Model 5600, Elitech Group Inc.) and was adjusted to 300–305 mmol/kg. Throughout the procedure, slices were bubbled with a 95% O_2_ and 5% CO_2_ mixture (carbogen). Slices were transferred to a submersion chamber (RC26G, Warner Instruments) that is continuously perfused with aCSF at a 2–3ml/min rate using a peristaltic pump (Miniplus 3, Gilson Inc.). All the recordings were performed at 33–34° C using an in-line heater (SH-27B, Warner Instrument Company) with a temperature controller (TC-324C, Warner Instrument Company), and the solutions were not recycled. Whole-cell voltage clamp or current clamp recordings were obtained from CA1 and CA3 neurons visualized via a 60X water immersion lens (LUMPLFLN60XW, N. A. = 1) attached to an upright microscope (Olympus BX51WI, Evident Scientific Inc.) equipped with DIC and a CCD camera (Retiga ELECTRO, Teledyne Technologies Inc). Neurons were patched using borosilicate glass pipettes (BF150–110-10HP, Sutter Instrument Company) with a pipette resistance (*R*_*p*_) of 3–5 MΩ, pulled using a Flaming/Brown pipette puller (P1000, Sutter Instrument Company).

### Whole-cell voltage clamp recordings in CA1

For whole-cell voltage clamp recordings, the pipette internal solution contained (in mM): 135 cesium-methanesulfonate, 10 HEPES, 8 NaCl, 5 QX-314 (HelloBio), 2 Mg-ATP, 1 EGTA, and 0.3 sodium-GTP hydrate (osmolarity = 290 mmol/kg). All chemicals were from Sigma unless otherwise specified. Voltage-clamp recordings were performed at a membrane potential of −70 mV. Spontaneous excitatory postsynaptic currents (sEPSCs) were obtained in the presence of 100μM picrotoxin (Hellobio). The series resistance (*R*_*s*_*)* was less than 25 MΩ in all the experiments, was not compensated, and was monitored offline. Recordings were discarded if the *R*_*s*_ changed by 25% during the experiment or if the holding current exceeded −250 pA. All recordings were obtained using an amplifier (Multiclamp 700B, Molecular Devices) and a digitizer (Digidata 1550B, Molecular Devices) interfaced with software (Clampex v11.3, Molecular Devices). The signals were filtered at 2 kHz using a Bessel filter and digitized at 10 kHz.

### Whole-cell current clamp recordings in CA1 and CA3

Two types of current clamp measurements were performed: 1) spontaneous excitatory postsynaptic potentials (sEPSPs) from the CA1 neurons, and 2) intrinsic properties of the CA3 neurons. Neurons in the CA1 region or between the CA3a and CA3b border are patched with a K^+^ containing internal solution that had the following components (in mM): 135 K-gluconate, 10 HEPES, 10 Na-phosphocreatine, 5 NaCl, 4 Mg-ATP, 2 MgCl2, 0.4 Na-GTP and 0.2 EGTA (osmolarity = 290 mmol/kg). All chemicals were from Sigma. The resting membrane potential (RMP, V_*rest*_) of the neurons was measured immediately after breaking in, and then we allowed 2–3 minutes for dialysis of the internal solution in the patched neurons. For CA1 experiments, sEPSPs were recorded for 5 minutes. CA1 recordings were performed at RMP (*I* = 0 mode) and discarded if the membrane potential depolarized below −60 mV. The bath perfusion aCSF for these experiments contained 100 μM picrotoxin. To assess the intrinsic properties of CA3 neurons, a membrane potential of −70 ± 2 mV was maintained by injecting a positive or negative current of 50 to −150 pA. Recordings were performed with aCSF solution containing 100 μM picrotoxin (Hellobio), 10 μM CNQX disodium salt (Hellobio) and 50 μM DL-APV sodium salt (Hellobio). The firing rate of the CA3 neurons was assessed by injecting a 25 pA depolarizing current step of 500 ms duration in increasing order (i.e., 0, 25, 50, 75, 100, 125, 150, 175, 200, and 0). The number of action potentials was counted at each step. For input resistance, a hyperpolarizing current of 500 ms duration was injected in −20 pA stepwise increments between 0 to −200 pA. The average change in voltage over the last 100 ms was taken as the steady-state response. The slope of a regression line fitted to the voltage-current relationship was used to calculate the overall input resistance. Action potential (AP) height and width were assessed by injecting a single 2 ms 1800 pA current pulse. AP height was measured by calculating the difference between the baseline and the peak amplitude. AP width was measured at 50% of the peak-to-baseline amplitude. All current clamp protocols were repeated at least 3 times, and the data were averaged. Junction potentials were not corrected. The signals were filtered at 2 kHz using a Bessel filter and digitized at 10 or 20 kHz.

### Data Analysis

All data are presented as mean ± SEM, with *n* = number of neurons. sEPSCs were detected using a template-matching algorithm in Easy Electrophysiology^®^ (Easy Electrophysiology Ltd). The detection limit was set to 3 times the root-mean-square of the peak-to-peak noise, which was typically around 4–6 pA. Burst(s) duration was excluded when calculating the sEPSC frequency. Intrinsic properties were analyzed using Clampfit v11.3 (Molecular Devices Inc.). Statistical analysis and graphs were generated using Prism v11 (GraphPad Inc.). Data were tested for normality (Shapiro-Wilk test), and based on the distribution, either the Brown-Forsythe test or the Kruskal-Wallis test was used. A *p-*value less than 0.05 was considered significant. Additional details regarding the statistical methods are presented in the Results and Figure Legends.

## Results

### Loss of *Doc2α* or *Syt7* results in increased CA3 burst generation

**Figure 1.**
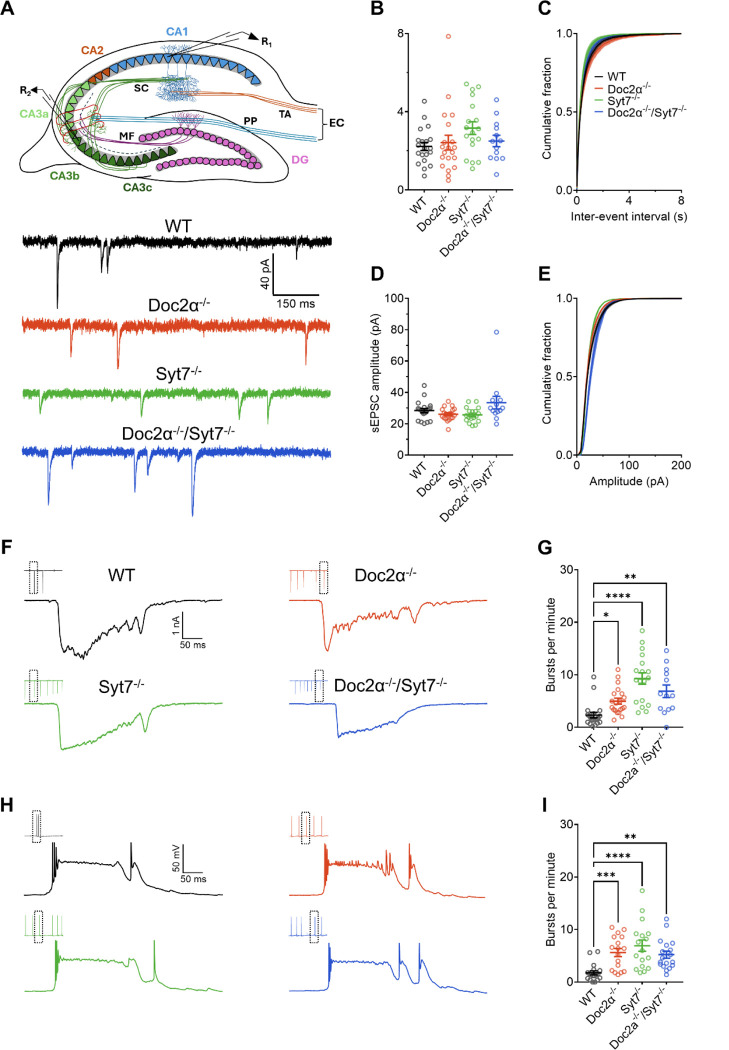
Increased spontaneous burst activity in the Schaffer collateral-CA1 circuit in *Doc2α*^*−/−*^*, Syt7*^*−/−*^ and *Doc2α*^*−/−*^*/Syt7*^*−/−*^ mice. A: Schematic diagram of the hippocampal slice preparation, highlighting the subfields, trisynaptic circuits and whole-cell recording electrodes placement (top panel); representative sEPSC traces (bottom panel). B-E: sEPSC data recorded from CA1 pyramidal neurons. B and C: sEPSC frequency and distribution, D and E indicate sEPSC amplitude and distribution. No statistical difference in sEPSC frequency or amplitude was observed (Kruskal-Wallis test with Dunn’s post-hoc correction). F-I: increased burst activity observed in CA1 neurons in slices from *Doc2α*^*−/−*^*, Syt7*^*−/−*^ and *Doc2α*^*−/−*^*/Syt7*^*−/−*^ mice. F and H: macroscopic views of representative bursts in voltage-clamp and current-clamp recordings, respectively, with insets showing 1 min traces. G and I: number of bursts per minute in voltage-clamp (*p < 0.05, **p < 0.01, ****p < 0.0001 vs WT, Kruskal-Wallis test with Dunn’s post-hoc correction) and current-clamp recordings (**p < 0.01, ***p < 0.001, ****p < 0.0001 vs WT, Kruskal-Wallis test with Dunn’s post-hoc correction), respectively. Note: Voltage-clamp and current-clamp recordings were obtained from different slice preparations. For voltage clamp recordings, n = 20 cells from 10 slices from 6 mice, WT; n = 20 cells from 10 slices from 6 mice, Doc2α^−/−^; n = 18 cells from 8 slices from 6 mice, Syt7^−/−^; n = 13 cells from 6 slices from 4 mice, Doc2α^−/−^/Syt7^−/−^. For current clamp recordings, n = 20 cells from 10 slices from 6 mice, WT; n = 20 cells from 11 slices from 7 mice, Doc2α^−/−^; n = 18 cells from 9 slices from 5 mice, Syt7^−/−^; n = 17 cells from 8 slices from 4 mice, Doc2α^−/−^/Syt7^−/−^. Data are expressed as mean ± SEM. CA: cornu ammonis; DG: dentate gyrus; EC: entorhinal cortex; PP: perforant pathway; MF: mossy fiber pathway; SC: Schaffer-collateral pathway; TA: temporoammonic pathway; R_1_: CA1 recording electrode placement for whole-cell voltage clamp; R_2_: CA3 recording electrode placement for whole-cell current clamp.

Given the numerous roles of Doc2α and Syt7 in the exocytosis of SVs and DCVs (Friedrich et al., 2008; Ramalingam et al., 2012; Houy et al., 2017; MacDougall et al., 2018; van Westen et al., 2021), we tested whether loss of these proteins affects excitatory network-mediated spontaneous activity in the mouse hippocampus. We carried out whole-cell voltage-clamp recordings from CA1 pyramidal neurons in disinhibited slice preparations (100 μM picrotoxin), a condition widely used to study CA3 burst activity (Buzsáki, 2015). The CA1 region receives major input from CA3 neurons via Schaffer collaterals (Fig. 1A). The activity of a single CA3 neuron is sufficient to trigger a burst within the subfield (Miles & Wong, 1983). Under these experimental conditions, hypersynchronous bursts are generated due to spontaneous activity in the CA3 subfield and then spread to the CA1 subfield via a feedforward mechanism (Shao & Dudek, 2009). In voltage-clamp experiments in CA1, we observed no significant change in sEPSC frequency or amplitude when comparing WT to *Doc2α-KO*, *Syt7-*KO, or DKO (Fig. 1A-E; p = 0.105 and p = 0.057, respectively; Kruskal-Wallis test with Dunn’s post-hoc correction; n = 20 cells from 10 slices from 6 mice, WT; n = 20 cells from 10 slices from 6 mice, Doc2α^−/−^; n = 18 cells from 8 slices from 6 mice, Syt7^−/−^; n = 13 cells from 6 slices from 4 mice, Doc2α^−/−^/Syt7^−/−^). However, we observed a striking increase in the number of bursts observed in the CA1 subfield; this increase was 2–4-fold in both knockouts, as well as the DKO, compared to the WT condition (Fig. 1F and G, *p* < 0.0001, Kruskal-Wallis test with Dunn’s post-hoc correction). We repeated these experiments in current-clamp mode and obtained similar results (Fig. 1H and I, *p* < 0.0001, Kruskal-Wallis test with Dunn’s post-hoc correction; n = 20 cells from 10 slices from 6 mice, WT; n = 20 cells from 11 slices from 7 mice, Doc2α^−/−^; n = 18 cells from 9 slices from 5 mice, Syt7^−/−^; n = 17 cells from 8 slices from 4 mice, Doc2α^−/−^/Syt7^−/−^). In current clamp, the bursts were characterized by 3–4 action potentials during the initial phase, followed by a large and prolonged depolarization phase, and are reminiscent of CSBs generated in CA3. Moreover, the burst activity is absent when a cut is made across the Schaffer collateral projection between the CA3 and CA1 (data not shown). These results, along with the voltage-clamp experiments (Fig. 1F-G), strongly indicate that the bursts originate in CA3. It is unclear whether these *in vitro* bursts correspond to enhanced *in vivo* SPW generation or represent pathological epileptiform activity.

### Intrinsic properties of CA3 pyramidal neurons are unchanged in *Doc2α-* and *Syt7-* KO mice

**Figure 2.**
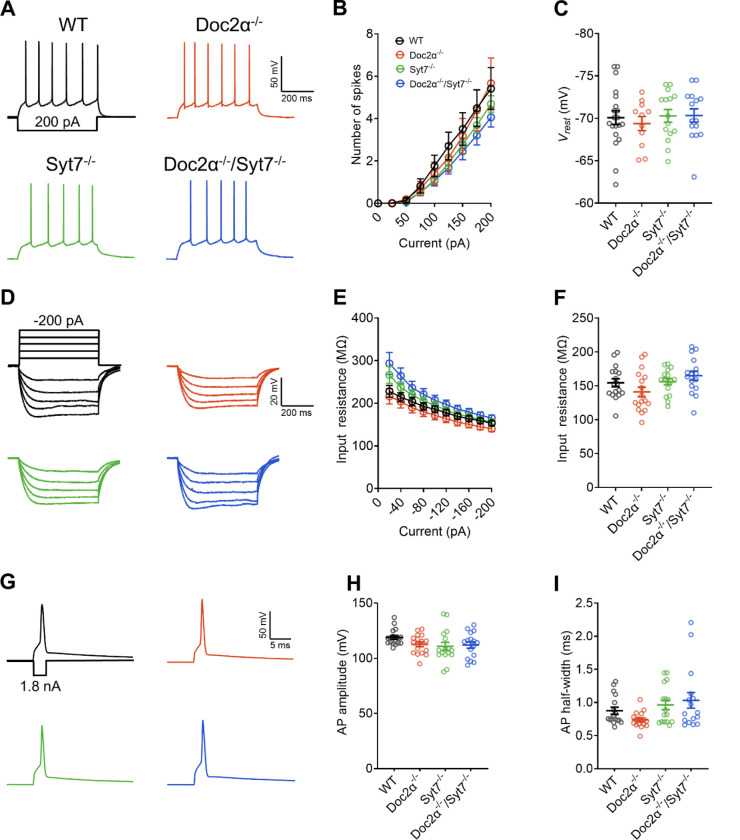
Intrinsic properties of CA3 pyramidal neurons are unaltered in *Doc2α*^*−/−*^*, Syt7*^*−/−*^ *and Doc2α*^*−/−*^*/Syt7*^*−/−*^ mice. A: Representative traces of neuronal firing upon injection of 200 pA of current. B and C: The firing rates at various current injection steps, and the resting membrane potential (V_rest_), are plotted. No statistically significant differences in resting membrane potential (*p* = 0.864, Brown-Forsythe test with Dunnett T3 post-hoc correction; n = 20 cells from 11 slices 7 mice, WT; n = 11 cells from 8 slices from 6 mice, Doc2α^−/−^; n = 15 cells from 8 slices from 6 mice, Syt7^−/−^; n = 15 cells from 9 slices from 5 mice, Doc2α^−/−^/Syt7^−/−^), or firing rate (*p* = 0.833, Brown-Forsythe test with Dunnett T3 post-hoc correction; n = 20 cells from 9 slices from 7 mice, WT; n = 11 cells from 10 slices from 6 mice, Doc2α^−/−^; n = 15 cells from 9 slices from 6 mice, Syt7^−/−^; n = 15 cells from 9 slices from 5 mice, Doc2α^−/−^/Syt7^−/−^) were observed. D: representative traces obtained during input resistance (R_input_) measurements. E and F indicate the R_input_ obtained at various current injection steps and the overall input resistance, respectively. No statistical difference in input resistance measurements (*p* = 0.0643, Brown-Forsythe test with Dunnett T3 post-hoc correction; n = 17 cells from 9 slices from 7 mice, WT; n = 17 cells from 10 slices from 6 mice, Doc2α^−/−^; n = 16 cells from 9 slices from 6 mice, Syt7^−/−^; n = 16 cells from 9 slices from 5 mice, Doc2α^−/−^/Syt7^−/−^). G: representative single action potential traces. H and I: quantitative measurements of action potential amplitude and half-width. No statistical difference was observed in AP height and half-width measurements (*p* = 0.059 and *p* = 0.104, respectively, Kruskal-Wallis test with Dunn’s post-hoc correction; n = 17 cells from 9 slices from 7 mice, WT; n = 17 cells from 10 slices from 6 mice, Doc2α^−/−^; n = 16 cells from 9 slices from 6 mice, Syt7^−/−^; n = 16 cells from 9 slices from 5 mice, Doc2α^−/−^/Syt7^−/−^).

Since increased burst generation could result from changes in the intrinsic properties of CA3 neurons (Pinsky & Rinzel, 1994), we examined these parameters via whole-cell current-clamp recordings. We patched neurons in the border between CA3a and CA3b, because of their higher recurrent collateral connectivity (Fig. 1A), which plays a role in burst initiation, and because numerous studies suggest that bursts originate in CA3a/b and spread to CA3c and CA1 via Schaffer collaterals (Buzsáki, 2015; Raus Balind et al., 2019). For these experiments, spontaneous synaptic activity was suppressed with 100 μM picrotoxin, 10 μM CNQX, and 50 μM APV, and a membrane potential of −70 mV was maintained throughout the recordings. In the first measurement, we found that the RMP (V_rest_) of the CA3 pyramidal neurons was unchanged (Fig. 2C, *p* = 0.864, Brown-Forsythe test with Dunnett T3 post-hoc correction; n = 20 cells from 11 slices 7 mice, WT; n = 11 cells from 8 slices from 6 mice, Doc2α^−/−^; n = 15 cells from 8 slices from 6 mice, Syt7^−/−^; n = 15 cells from 9 slices from 5 mice, Doc2α^−/−^/Syt7^−/−^). We then assessed the firing rate of CA3 neurons by injecting a depolarizing current in 25 pA increments, from 0 to 200 pA. We did not observe any significant changes in the mean firing rate at various current steps (Fig. 2A and B, *p* = 0.833, Brown-Forsythe test with Dunnett T3 post-hoc correction; n = 20 cells from 9 slices from 7 mice, WT; n = 11 cells from 10 slices from 6 mice, Doc2α^−/−^; n = 15 cells from 9 slices from 6 mice, Syt7^−/−^; n = 15 cells from 9 slices from 5 mice, Doc2α^−/−^/Syt7^−/−^). We then examined the input resistance (R_input_) of these neurons by injecting hyperpolarizing current in increments of 20 pA, from 0 to −200 pA. The overall R_input_ (Fig. 2D-F, *p* = 0.0643, Brown-Forsythe test with Dunnett T3 post-hoc correction; n = 17 cells from 9 slices from 7 mice, WT; n = 17 cells from 10 slices from 6 mice, Doc2α^−/−^; n = 16 cells from 9 slices from 6 mice, Syt7^−/−^; n = 16 cells from 9 slices from 5 mice, Doc2α^−/−^/Syt7^−/−^), at each current step, was the same between the groups. The action potential half-width and amplitude are also unchanged in both the KOs (Fig. 2G-I, *p* = 0.059 and *p* = 0.104, respectively, Kruskal-Wallis test with Dunn’s post-hoc correction; n = 17 cells from 9 slices from 7 mice, WT; n = 17 cells from 10 slices from 6 mice, Doc2α^−/−^; n = 16 cells from 9 slices from 6 mice, Syt7^−/−^; n = 16 cells from 9 slices from 5 mice, Doc2α^−/−^/Syt7^−/−^). Overall, we did not observe significant changes in the intrinsic properties of CA3 pyramidal neurons, suggesting that the increases in burst activity in the KOs could result from alterations in SV or DCV release.

## Discussion

In this study, we used KO mice to probe the potential functions of *Doc2α* and *Syt7* in spontaneous excitatory network activity in the hippocampus. We found that loss of either of these presynaptic proteins results in increased burst generation in the hippocampal CA3 subfield, without affecting sEPSC frequency, in slices from juvenile mice (P15–21). Our whole-cell current-clamp measurements show that the observed phenotype is not due to changes in the intrinsic properties of CA3 pyramidal neurons, suggesting alterations in SV or DCV release. To the best of our knowledge, this is the first study to report the effect of *Doc2α-* and *Syt7-* KO on burst generation in the hippocampal CA3 subfield.

### The CA3 subfield and burst generation

The hippocampal CA3 subfield is a critical region for associative learning and memory (Joo & Frank, 2018). There are three main inputs to the CA3: i) projections from the entorhinal cortex (perforant pathway), ii) projections from dentate granule cells (mossy fiber pathway), and iii) associative-commissural projections due to RCs (Fig. 1A). All three inputs are spatially segregated into different layers of the CA3 subfields - a, b and c - with CA3a and CA3c being distal and proximal to the dentate gyrus (DG). CA3a and CA3b axons ramify extensively and form RCs, generating CSBs leading to network-level activity termed sharp waves (SPWs). The SPWs propagate to CA3c, then to CA1, and induce sharp wave ripples (SPW-Rs). Generation of SPWs and SPW-Rs is prominent during rest and sleep and is a critical physiological mechanism for information transfer and memory consolidation (Buzsáki, 2015; Rebola et al., 2017). CA3 burst generation is a complex phenomenon that requires tightly controlled spatiotemporal integration. These bursts are generated by intrinsic mechanisms, network activity, or a combination of both (Zeldenrust et al., 2018). A plethora of cellular and molecular mechanisms have been proposed to modulate this synaptic integration (Buzsáki, 2015). Recent evidence suggests that CA3 neurons generate bursts due to Ca^2+^ spikes that produce slow afterdepolarization in the soma by prolonging dendritic plateau potentials (Raus Balind et al., 2019). These plateau potentials play a role in the induction of behavioral time-scale plasticity (BTSP) in the CA3 (Li et al., 2024; Madar et al., 2025; Magee, 2026). In addition, imbalances in burst generation can lead to pathological epileptiform activity as seen in neurological or neurodevelopmental disorders (Goldberg & Coulter, 2013; Buzsáki, 2015).

### CA3 burst activity is increased in Doc2α and Syt7 KO hippocampi independent of alterations in synaptic transmission

We reiterate that Doc2 proteins act as Ca^2+^ sensors that regulate the spontaneous release of SVs, also called miniature neurotransmitter release or minis, in acute slices and cultured hippocampal neurons; their relative contribution to minis in the hippocampus depends on the specific synapse under study (Groffen et al., 2010; Ramirez et al., 2017; Courtney et al., 2018; Khan & Regehr, 2020; Wang et al., 2023). However, loss of Doc2α-triggered minis is unlikely to regulate bursting activity, since paired recordings showed that the RCs of CA3 neurons have unitary EPSPs of ~0.56 mV, which is insufficient to trigger an action potential in the postsynaptic cell (Guzman et al., 2016). In addition, there is no clear evidence for a role of Syt7 in the regulation of minis in wild-type neurons (Chen et al., 2017; Luo & Südhof, 2017). These findings argue against a role for minis in the bursting behavior described in the current study.

Although AR is a common mechanism that is supported by both Doc2α and Syt7, it is unlikely that changes in this slow mode of transmission underlie the observed changes in burst activity. First, AR of quanta in excitatory synapses would maintain the post-synaptic neuron in a depolarized state for prolonged periods, enhancing the spiking window and temporal precision of AP firing (Iremonger & Bains, 2007; Evstratova et al., 2014). Knocking out proteins important for AR therefore should reduce excitation, but we observed the opposite: increased burst generation. Second, studies involving AR measurements typically use 2 mM Ca^2+^ or higher, and recordings are performed at RT to enhance the asynchronous quanta. The increased burst phenotype observed in our study is seen under near-physiological conditions (i.e., 1.2 mM Ca^2+^ and 33–34 °C). No studies have carefully evaluated quantal AR, binned over time, at hippocampal excitatory synapses, but cumulative charge transfer analysis suggests that only low levels of this form of slow transmission occurs in the hippocampus (Yao et al., 2011; Wu et al., 2024), which is markedly lower as compared to other brain regions, including a number of distinct types of synapses in the cerebellum (granule cells to stellate cells etc). Third, the CA3 RCs also make it difficult to quantify AR using the traditional field-stimulation approach, because of recurrent network activity. Nevertheless, we measured AR in the SC-CA1 (as a proxy for CA3-CA3 RCs) in Doc2α and Syt7 KO hippocampi and found no significant difference in AR quanta at this specific synapse under near-physiological conditions (data not shown). Hence, we argue that the burst activity in the CA3 subfield is unlikely to be due to changes in AR, but this cannot be ruled out. Optical methods, such as iGluSnFR-based approaches, might make it possible to overcome technical limitations and enable analysis of AR within the RCs of the CA3 region, but at present, this approach suffers from limited temporal resolution.

### An alternate hypothesis

In addition to neurotransmitter release, both Doc2α and Syt7 regulate peptide release from DCVs (MacDougall et al., 2018). Neuropeptides secreted by the inputs to CA3 regulate burst firing of pyramidal neurons. Namely, neuropeptide-Y (NPY) and C-type natriuretic peptide (CNP) suppress the generation of sharp waves in the CA3 subfield by decreasing feed-forward excitatory transmission (Klapstein & Colmers, 1993; Decker et al., 2009; Pollali & Draguhn, 2025). The CA3 subfield layers, *stratum radiatum* and *stratum oriens*, are enriched in NPY receptors (Klapstein & Colmers, 1993). NPY is secreted by DG and CA3 interneurons that often co-express somatostatin (Pelkey et al., 2017). NPY suppresses glutamate release by reducing Ca^2+^ influx in nerve terminals (Colmers et al., 1988). It is important to note that NPY does not alter the passive and active membrane properties of CA3 neurons.

NPY is of particular interest since mossy fiber terminals, the major inputs to the CA3, are also rich in NPY-bearing DCVs (Henze et al., 2000). However, a recent study showed that the selective knockout of Syt7, induced via AAV injection in the DG (to disrupt expression in mossy fibers), reduced the likelihood of CA3 population activity *in vivo* by reducing spike transfer from DG to CA3 (Marneffe et al., 2026), which would seem to be inconsistent with our observations. Rather, Marneffe et al. (2000) proposed that abrogation of facilitation was the underlying mechanism. However, the authors estimate a ~60% loss of Syt7 in dentate granule cells (due to incomplete coverage of the virally-induced knockout), with DG and CA3 interneurons spared. So, it remains possible that loss of Syt7 disrupts the release of burst-suppressing neuropeptides such as NPY, and thereby promotes burst activity.

Extending this hypothesis to Doc2 proteins, we reiterate that, at the mRNA level, dentate granule cells express both *Doc2α* and *Doc2β*, whereas CA3 cells express only *Doc2α* (Verhage et al., 1997), but it is not known if both isoforms are translated, and isoform-specific antibodies are not currently available. We did not observe the burst phenotype in *Doc2β* KOs (data not shown), so either Doc2β is not expressed, or it fails to regulate a neuropeptide that controls bursting, or our hypothesis regarding mossy fiber control of bursting activity is incorrect. In addition, whether Doc2α regulates neuropeptide release from neurons has been called into question in studies using over-expressed NPY fusion proteins in cultured hippocampal neurons (van Westen et al., 2021). This will require careful re-examination, as Doc2α has been shown to regulate the release of dense core vesicles in neuroendocrine and non-neuronal cells (Friedrich et al., 2008; Ramalingam et al., 2012; Houy et al., 2017; MacDougall et al., 2018).

While there are arguments for and against the inhibitory neuropeptide hypothesis, we reiterate that this remains a leading model, since the only remaining untested function that is conserved between Doc2α and Syt7 - albeit across different cell types - is the control of DCV release. In this light, it is notable that the bursting phenotype was not additive in the CA3 subfield from Doc2α and Syt7 double KO mice. This finding is consistent with the idea that both proteins act in the same pathway. Further testing of the DCV hypothesis will require measurements of neuropeptide release within the CA3 region of *Doc2α-* and *Syt7-* knockout mice, perhaps via development of G-protein-coupled activation-based (GRABs) sensor for NPY and other candidate peptides.

### Limitations of the study

The current study utilized juvenile mice, so it is possible that the phenotype we observed is limited to this developmental period. In this light, we note that Sholl analysis of dentate granule cells showed increased neurite branching in *Doc2α* KO mice (Wang et al., 2023). Increased branching of axons and dendrites during postnatal development is one mechanism underlying CA3 hyperexcitability (Gómez-Di Cesare et al., 1997). Notably, the observed increase in branching complexity was absent in adult mice. We also did not characterize the behavioral phenotypes exhibited by the *Doc2α-* and *Syt7-* KO mice. Although both the KOs are viable and appear normal, recent reports suggest they may have developmental issues (Shen et al., 2020; Wang et al., 2023). For example, loss of Doc2α has been proposed to cause autism spectrum-like disorder in mice, in the same age group as the mice used in our study (Wang et al., 2023). Finally, it is not yet known whether there is an increase in generation of SPWs in *Doc2α-* and *Syt7-* KO mice *in vivo*, or whether the increased burst activity interferes with memory-dependent tasks. These are important areas of inquiry in future studies.

In conclusion, using juvenile *Doc2α-* and *Syt7-* KO mice, we uncover a novel burst-generating phenotype in the hippocampal CA3 subfield. Our study has implications for understanding the roles of these presynaptic proteins in regulating excitatory transmission in hippocampal circuits and their associated physiological functions.

## Data Availability

All the raw data is available on Dryad, a publicly accessible repository. https://doi.org/10.5061/dryad.wpzgmsc50

## Key points

- CA3 burst activity is critical for associative learning and memory consolidation
- Doc2α and Syt7 are high-affinity Ca^2+^ sensors with similar kinetic properties and are expressed in the hippocampus
- Doc2α and Syt7 support various modes of neurotransmitter release and presynaptic plasticity
- Both Doc2α and Syt7 are also involved in the exocytosis of dense-core vesicles in neuroendocrine cells
- Loss of Doc2α and Syt7 results in increased CA3 burst activity without a change in the intrinsic properties of CA3 neurons
- Our results suggest a novel role for Doc2α and Syt7 in regulating network excitatory activity
